# Indigenous *Trichoderma* species multi-mechanistic antagonism against *Fusarium* root disease in *Coffea arabica*: volatile-mediated inhibition, morphological, and molecular profiling

**DOI:** 10.3389/fmicb.2026.1899216

**Published:** 2026-09-10

**Authors:** Shroog M. Al-Shahrani, Abdulaziz A. Al-Askar, Kevin D. Hyde, Fatimah Al-Otibi

**Affiliations:** 1 Department of Botany and Microbiology, College of Science, King Saud University, Riyadh, Saudi Arabia; 2 Center of Excellence in Fungal Diversity, Mae Fah Luang University, Chiang Rai, Thailand

**Keywords:** *Fusarium* sp., GC–MS analysis, internal transcribed spacers, root disease of *Coffea arabica*, *Trichoderma* sp., volatile organic compounds

## Abstract

**Introduction:**

There is a rising prevalence of root pathogens of the genus Fusarium on coffee (*Coffea arabica*) farms in Saudi Arabia. The current study aimed to explore the antagonistic mechanisms involved in the interaction between four *Trichoderma* spp. isolated from coffee rhizospheres and two different strains of *Fusarium* spp., namely *Fusarium oxysporum and Fusarium brachygibbosum.* The main hypothesis is that the mechanism of action for the *Trichoderma-Fusarium* interaction involves three distinct and concurrent mechanisms, which include: (i) spatial competition due to differences in growth rates, (ii) production of antifungal metabolites, and (iii) Volatile Organic Compounds (VOC) targeting selected cellular targets within fungi.

**Methods:**

The root system and rhizosphere soil of coffee plants from localities in Al-Baha and Jazan regions (May and August 2023) were examined for the presence of *Trichoderma* and *Fusarium* species. Six *Trichoderma* and *Fusarium* isolates were identified by molecular analysis using ITS sequencing. The antagonistic activities of the identified isolates were tested using dual culture assays, incubated at 27 °C for 7 days. The percent mycelial inhibition was calculated. VOCs released by the four *Trichoderma* species were extracted with ethyl acetate and analysed using GC–MS. The inhibition of fungal growth was explained by hierarchical regression, which partitioned the variance in inhibition by the growth rate of the fungi, VOC diversity, and specific compounds.

**Results:**

The *Trichoderma* strains showed 2.6–3.2x faster radial growth rate (15–19 mm/day) compared to *Fusarium* strains (5.9–7.9 mm/day). *Trichoderma harzianum* 404.1 and *Trichoderma asperellum* J-3 were found to produce the highest inhibition of *F. brachygibbosum* at 77.4 and 77.6%, respectively (*p* < 0.001). This was better than inhibition by the other two strains, such as *Trichoderma longibrachiatum* and *Trichoderma koningii,* which inhibited at 73.1 and 75.1%, respectively. Sixty-nine unique VOCs were identified in the four strains using GC–MS. The compounds of mechanism-related relevance included 1,8-cineole (eucalyptol), caryophyllene oxide, 6-pentyl-2H-pyran-2-one (6-PP), and 2-phenylethanol. *T. harzianum* 404.1 showed maximum VOC production, with 20 types, whereas *T. asperellum* J-3 produced only caryophyllene oxide. Therefore, our proposed mechanistic model involved all three steps: competition of *Trichoderma* spp. with pathogens for nutrition, emission of different VOCs to affect fungi on different fronts, and release of metabolites such as hexanedioic acid esters and phthalate derivatives, having mycolytic potential.

**Conclusion:**

These findings provide a mechanistic framework for the development of species-specific *Trichoderma-*based bio-fungicides and for field validation under Saudi Arabian coffee-producing conditions.

## 1 Introduction

Coffee is, after petroleum, the world’s second most traded commodity. Trade in coffee provides income and means of living to over 125 million individuals worldwide. Of the 130 species within the genus *Coffea*, two species—*Coffea arabica and Coffea canephora* – are cultivated by humans to be used as food. They constitute around 70 and 30% of global coffee production (de Sousa et al.., 2022). Of these two, *Coffea arabica* is the most cultivated species of coffee and represents about 80% of the coffee cultivation area in the world (Ge et al., 2023). *C. arabica* is widely cultivated and highly prized by coffee consumers for its far superior flavor to that provided by *Coffea canephora* (robusta) (Hilda and Syafiruddin, 2021). The origin of *Coffea arabica* is in Africa, and specifically, Ethiopia is considered to be the birthplace of Arabica coffee (Alemu, 2025). In southwestern Saudi Arabia, as well as in Yemen, where coffee was first cultivated centuries ago, the geography is close to the region in Ethiopia where coffee first grew. Currently, coffee is cultivated in several provinces in Saudi Arabia, to wit, Al Baha, Asir, Jazan, and Najran (Alsubaie et al., 2024). Many are the economic objectives of Saudi Vision 2030, but above all, a very important one is the goal to develop the coffee industry. The Saudi coffee market in 2021 alone reached the value of USD 1.58 billion and is projected by Ismail et al. (2025) to reach a value of approximately USD 2.22 billion by the end of the year 2028. By the end of the year 2026, an increase of 1.2 million coffee trees will be cultivated as a component of the overall agricultural development objective (Salian, 2023).

Diseases affect coffee production worldwide and specifically in coffee-growing areas of Saudi Arabia, with fungal diseases being the most prominent (Bracken et al., 2023). Of these fungal diseases, *Fusarium* diseases have become a major global threat to key crops worldwide over the last 100 years, including wheat, barley, maize, bananas, cotton, and coffee, often causing severe socio-economic consequences (Corbu et al., 2023). The majority of *C. arabica* varieties are susceptible to infection by various *Fusarium* spp., causing wilt and dry root rot in coffee trees (Al-Faifi et al., 2022). *Fusarium* diseases have emerged as an imminent danger to coffee production in various regions around the world, particularly in Eastern Africa where Coffee Wilt Disease (CWD) caused by various types of *Fusarium* fungi has brought great economic damage (Muller et al., 2004; Tshilenge-Djim et al., 2016). Although *Hemileia vastatrix* (Coffee Leaf Rust) is the most economically devastating pathogen of *Coffea arabica* around the world, *Fusarium* wilt and dry root rot are emerging as major concerns in various coffee-producing countries (Al-Faifi et al., 2022; Peck and Boa, 2023). In Uganda, CWD caused by *Fusarium xylarioides* has led to destruction of more than 1.5 million coffee plants from 1993 to 2003, with an annual economic loss of USD 20–30 million (Peck and Boa, 2023). In Saudi Arabia, recent studies have shown the presence of various Fusarium species causing symptoms of wilts and root rots on coffee farms with incidence levels ranging from 15 to 30% (Al-Faifi et al., 2022; Ismail et al., 2025; Alhudaib et al.., 2023).

In contrast, the use of chemical compounds to control plant diseases has several limitations, such as resistance of pathogens to fungicides, which has been reported worldwide (Pereira et al., 2020). In addition, the broad use of fungicides can affect non-target organisms and is generally discouraged (Pereira et al., 2020). Biocontrol methods using natural pests’ natural enemies are inherently safe and present low risk of pest resistance (Xiao et al., 2023). *Trichoderma* species are one of the most extensively utilized biocontrol agents because of their antagonistic action against many plant diseases. *Trichoderma* spp. are efficient biocontrol agents due to their fast growth and high sporulation (Rolim et al., 2019). For effective biocontrol, antagonistic fungi are best isolated from the roots or rhizosphere of the crops of interest, as these fungi are ‘rhizosphere competent’ and better to use in the field conditions (Cook, 1993). There are several mechanisms by which *Trichoderma* spp. control plant pathogens, including the production of compounds that are antibiosis, either in the form of volatile compounds or non-volatile antibiotics, mycoparasitism that involves the use of lytic enzymes, and competition for space and nutrients (Benítez et al., 2004; Vizcaíno et al., 2005; Sánchez et al., 2007). The study of the volatile organic compounds (VOCs) produced by *Trichoderma* is a new area of research with potential agricultural applications (Elouark et al., 2025). A total of 480 VOCs has been reported to be produced by the VOCs of *Trichoderma* spp. (Guo et al., 2019). The bioactive VOCs produced by *Trichoderma* spp. have several effects on plant pathogens, on plant resistance, and on plant growth (Lee et al., 2016; Kottb et al., 2015; Li et al., 2018).

Three hypotheses were proposed for testing: (H₁) There are *Trichoderma* spp., isolated from the coffee rhizosphere, that show VOC fingerprints in a species-specific way, which are associated with differential antagonism against *Fusarium*; (H₂) the success of inhibition relies mainly on growth advantage, rather than a diverse VOC production capability; (H₃) there are some VOCs, with known mechanisms of antifungal actions, that show species-specific distribution. The following objectives were considered: (i) Isolation and identification of *Trichoderma* from the Saudi coffee roots/rhizosphere; (ii) Quantifying the *in vitro* antagonism against *F. oxysporum* and *F. brachygibbosum*; (iii) Characterization of VOCs by GC–MS analysis.

## 2 Materials and methods

### 2.1 Sample collection

The study was conducted in the southwestern region of Saudi Arabia, which is characterized by its height and mountainous nature, providing suitable climatic conditions for coffee cultivation. Samples were obtained from three independent farms representing different geographical locations within this region. The first sampling site (Farm 1) was in Jabal Shada Al-A’la in the Al-Baha region (19°50′35.4″N, 41°18′39.0″E). The second site (Farm 2) was situated in Jabal Shada Al-Asfal within the Al-Baha province (19°44′26.8″N, 41°21′19.2″E). The third site (Farm 3) is located in the Al-Shat Al-Kabeer area of Wadi Al-Ain, Jazan region (17°20′58.8″N 43°12′18.4″E). Samples were obtained at each farm from five randomly selected coffee trees exhibiting wilt signs, with a minimum distance of 10 meters between trees. Three soil samples (250 g each) from the rhizosphere of each tree were taken at a depth of 10–15 cm, as well as three root samples (about 5 cm in length). The samples were pooled by farm to create representative composite samples. The samples were collected in May 2023 and August 2023.

Samples of *Trichoderma* and *Fusarium* were isolated from rhizosphere soil and roots of wilt-infected coffee plants. For the rhizosphere samples, 250 g of soil was excavated to a depth of 10–15 cm from each coffee plant and collected in sterilized polyethylene bags. Root samples of coffee trees with abundant wilting symptoms were collected, cut into small pieces, and individually stored in plastic bags. All samples were transported to the microbiology laboratory of King Saud University and stored at 4 °C for analysis.

### 2.2 Isolation and purification of *Trichoderma* and *Fusarium* spp.

10 g of soil samples were suspended in 90 mL of sterile distilled water to provide a soil suspension, which was combined in a rotary shaker (Thermo Fisher Scientific Inc., Waltham, MA, United States) at 100 rpm for 10 min. To isolate the *Trichoderma* and *Fusarium* colonies from the soil, a soil serial dilution was prepared using the soil suspension. To provide an initial dilution of 10%, 1 mL of soil suspension was added to 9 mL of distilled water. Subsequent dilutions were prepared at a dilution factor of 1:10 and labeled as 10^2^, 10^3^, 10^4^, and 10^5^. 1 mL of each dilution was pipetted into a Potato Dextrose Agar (PDA) Petri dish, supplemented with chloramphenicol (Sigma-Aldrich, St. Louis, MI, United States). For each species, three replicate cultures were prepared from each dilution (Sehim et al., 2023).

The roots were washed gently with running tap water to remove all soil particles. From each sample, 1 cm segments of root slices were immersed in distilled water (2 min.), surface-sterilized by immersing them in sodium hypochlorite (BDH Chemicals, Poole, United Kingdom) (7% available chlorine; 1–2 min.), then rinsed with distilled water three times. They were then allowed to dry on sterile filter paper for 10 min in a sterile laminar flow chamber. Segments were placed horizontally on the PDA supplemented with chloramphenicol. Isolations of both *Trichoderma* and *Fusarium* were performed from single spores to achieve pure culture. A sterile needle was employed to pick up one conidium or spore and transferred to a fresh PDA plate to check under the microscope. All plates were incubated at 27 °C for 7 days until the fungus grew. In order to obtain a pure culture from all samples, the visible fungal colonies were isolated onto PDA media after being identified using macromorphological features similar to the primary selection of *Trichoderma* and *Fusarium* colonies, and the daily growth rate in the plates was measured. Before DNA analysis, purity of the isolate was confirmed microscopically.

### 2.3 Characterization of *Trichoderma* and *Fusarium* spp.

For 7 days, a pure culture of the suspected *Trichoderma* isolates was cultivated on PDA medium and incubated at 27 ± 1 °C. Macroscopic features such as sporulation on PDA media, pigment production, and the color of the colony surface on both the upper and lower sides, and the texture of the colony, were noted. Microscopic details of each isolate, including the characteristics of the conidiophores and the shape and color of the conidia, were examined on glass slides under light microscopes. Species identification was based on the previously reported morphological and taxonomic keys (Gams and Bissett, 1998; Siddiquee, 2017).

The *Fusarium* isolates were initially identified based on their cultural and microscopic characteristics following the taxonomic keys described for the genus *Fusarium*. Colony morphology was observed on PDA after incubation at 27 ± 1 °C for 7 days, from both the top and bottom. Microscopic structures, including macroconidia, microconidia, and chlamydospores, were examined and compared with the taxonomic descriptions previously reported (Leslie and Summerell, 2006).

### 2.4 Molecular identification of *Trichoderma* and *Fusarium* spp.

The crude fungal genomic DNA extraction comprised collecting pure fungal colonies from each sample from culture plates into a screw-cap microcentrifuge tube, where the colonies were pelleted by centrifugation for 1 min. at 13,000 rpm, and the supernatant was discarded. The pre-treatment was carried out using the PrepMan™ Ultra Reagent (Thermo Fisher Scientific Inc., Waltham, MA, United States) boiling method to prepare crude genomic DNA (gDNA). In short, 100 μL of PrepMan™ Reagent was mixed with the sample tube and vortexed for 10–30 s. Tubes were then heated for 10 min in a Digital Heating Shaking Drybath (Thermo Fisher Scientific Inc., Waltham, MA, United States) at 100 °C and then cooled to room temperature for 2 min. After extraction, the samples were centrifuged for 2 min at maximum speed, with the supernatant containing the crude genomic DNA. 50 μL of the supernatant was transferred into a labeled, new tube. 1.5 ~ 2 μL of supernatant was used as the PCR template.

The Internal Transcribed Spacers (ITS) region was amplified using the primers ITS1 5′ (TCC GTA GGT GAA CCT GCG G) 3′ and ITS4 5′ (TCC TCC GCT TAT TGA TAT GC) 3′ for the PCR of the ITS region (Integrated DNA Technologies, Coralville, IA, United States). PCR reactions (total volume 20 μL) were assembled using 10 μL of PCR master mix 2x (Axen™ H Taq PCR Master Mix), 1 μL of each primer pair, 7 μL of sterile distilled water (HPLC grade), and 2 μL template (≈20 ng/μL). The thermocycling program used was an initial denaturation at 95 °C for 2 min (1 cycle), 31 cycles of denaturation at 95 °C for 30 s, annealing at 57 °C for 30 s, and elongation at 72 °C for 1 min, and the final stabilization at 72 °C for 10 min (1 cycle). PCR products were visualized on ethidium bromide-stained 1.5% agarose gels with 1 × Tris-Borate-EDTA (TBE) buffer (Sigma-Aldrich, St. Louis, MI, United States). The nuclear DNA extraction, amplification of ITS1-5.8S rDNA sequencing, and purification of the amplified PCR products from fungal isolates were performed at MACROGEN (Seoul, South Korea).^1^

We cleaned and assembled the obtained DNA sequences using Geneious Prime® 2024.0.7 software. After that, the acquired ITS sequences were compared with reference sequences deposited in the GenBank database using the Basic Local Alignment Search Tool (BLAST) in GenBank.^2^

The phylogenetic trees of the *Trichoderma* and *Fusarium* strains were constructed by matching the fungal isolates’ ITS sequences with the available GenBank references using the software tool MEGA 11 (Molecular Evolutionary Genetics Analysis). Clustal W was used to perform the alignment, which followed typical settings. The NJ (Neighbor-Joining) technique was used to create trees, together with the Kimura 2 parameter substitution model. 1,000 replications were employed during bootstrapping. The analysis also includes outgroup DNA from closely related species. The maximum composite likelihood approach was used to calculate evolutionary distances. All alignment points, including gap alignments, were deleted (full deletion technique). The trees were shown and edited using FigTree v1.4.4.^3^

### 2.5 Antagonistic activity of *Trichoderma* isolates against *F. oxysporum* and *F. brachygibbosum*

Evaluation of the antagonistic potency of *Trichoderma* spp. against *Fusarium* strains was performed using a dual culture technique. Mycelial discs (9 mm in diameter) were cut from the margins of a 5-day-old culture of each strain using a sterile cork borer. Mycelial discs of both antagonistic strains and fungal pathogens *F. oxysporum* and *F. brachygibbosum* were inoculated separately over freshly prepared PDA medium and placed 3 cm away from both edges of the plates. The control PDA plate was treated using the same method, but without *Trichoderma*. Three replicates of the experiment were carried out. All plates were left at 27 °C for 7 days. The percentage of radial growth inhibition of *Fusarium* sp. was measured in comparison to the control plates and computed using the following formula (Kamala and Indira, 2011):


$$\text{Radial Growth Inhibition}\%=\frac{C-T}{C}\times 100$$

Where “C” is the radial growth of *Fusarium* sp. (pathogen) in the control plate and “T” is the radial growth of *Fusarium* sp. (pathogen) in the presence of *Trichoderma* spp.

On day 7, the interface between *Trichoderma* and *Fusarium* colonies was examined microscopically at 40 × and 1,000 magnification. Mycoparasitism was assessed using a 0–4 scale (Table 1) (Tkalenko et al., 2020). Three replicate plates for each isolate pair were examined, and five random fields from each plate were scored.

**Table 1:** Mycoparasitism scoring.

Score	Description
0	No interaction between the two fungi, and the hyphae of *Trichoderma* and *Fusarium* were completely separate
1	The hyphae of *Trichoderma* were in contact with the hyphae of *Fusarium*, but there was no coiling
2	Coiling of the hyphae of *Trichoderma* around the hyphae of *Fusarium* in less than 25% of the examined fields
3	Coiling of the hyphae of *Trichoderma* around the hyphae of *Fusarium* in 25–50% of the examined fields, and the *Trichoderma* hyphae were swollen
4	Extensive coiling of the hyphae of *Trichoderma* around the hyphae of *Fusarium* in more than 50% of the examined fields, and there were penetrative structures associated with evidence of degradation of the hyphae of *Fusarium*.

### 2.6 Evaluation of volatile metabolites of *Trichoderma* spp. by gas chromatography mass spectrometry analysis (GC–MS)

Initially, 9 mm mycelial disc from each of the four actively growing *Trichoderma* isolates was inoculated into 250 mL of potato dextrose broth and incubated for 7 days at 25 °C. Following this incubation, the potato dextrose broth containing the developed mycelia underwent centrifugation for 15 min at 9,000 rpm after being filtered twice through Whatman no. 1 filter paper. The resulting filtrates were combined with an equal volume of ethyl acetate solvent (Sigma-Aldrich, St. Louis, MI, United States) and agitated overnight at 25 °C and 150 rpm to extract the volatile metabolites. Subsequently, a separating funnel was utilized to isolate the solvent phase. To concentrate the solution containing volatile organic compounds, a rotary evaporator was employed until complete evaporation of the solvent occurred. Finally, to dilute the end product, 2 milliliters of ethyl acetate were incorporated (Lakhdari et al., 2023).

The analysis of crude extracts from *Trichoderma* spp. was performed utilizing Shimadzu Gas Chromatography (Kyoto, Japan) equipped with Turbo Mass Gold mass detection, which included an Elite −1 column (100% Dimethyl Polysiloxane), measuring 30 m x 0.25 mm ID x one mM df. Potential VOCs from *Trichoderma* spp. were identified under specific conditions: 1.0 μL of the final ethyl acetate extract was injected, with helium serving as the carrier gas at a flow rate of 1 mL/min; the oven temperature was programmed to increase from 110 °C (held for 2 min) to 280 °C (held for 9 min), while the injector temperature was set at 250 °C, culminating in a total GC analysis time of 45 min. A computer software program facilitated the identification of key VOCs present in the samples, comparing results against both version 5.1 of Turbo Mass and the National Institute of Standards Technology (NIST) library database. The GC–MS analysis was conducted by the Central Research Laboratory at King Saud University’s Female Center for Medical Studies and Scientific Section (Lakhdari et al., 2023).

VOC peak areas were normalized to the total ion chromatogram (TIC) area to express their relative abundance (%). The relative abundance of individual VOCs with known antifungal bioactivities was compared between *Trichoderma* spp. by one-way ANOVA. The relative abundance of individual VOCs of interest was also correlated with percentage inhibition of *F. oxysporum* and *F. brachygibbosum* growth by Pearson correlation coefficients to identify individual VOCs that may function as mechanism-specific potency markers.

### 2.7 Statistical analyses

Statistical analysis was performed using IBM SPSS Statistics version 26 (IBM Corp., Armonk, NY, USA). The experiment followed a completely randomized design (CRD) with three replicates per treatment. Data are presented as mean ± SD. A Chi-square test (χ^2^) of independence was used to assess whether mycelial growth inhibition over 7 days was dependent on *Trichoderma* treatments. A paired-sample t-test was employed to compare pathogen growth between the dual culture treatments and their respective controls. Significance levels were reported at *p* < 0.05.

To partition the variance in *Fusarium* inhibition between different mechanisms of action, we performed multiple linear regression with *Fusarium* inhibition percentage as the dependent variable, and the following independent variables: (1) *Trichoderma* growth rate (mm/day), (2) total number of VOCs, (3) number of VOCs with known antifungal activity, and (4) presence/absence of certain mechanism class compounds (terpenoids, aromatic alcohols, pyrones, and phthalates). We used stepwise regression for variable selection, with an entry *p*-value < 0.05. All assumptions of linear regression (normal distribution of residuals, homoscedasticity, and independence) were checked.

## 3 Results

### 3.1 Isolation and purification

Eight different *Trichoderma* isolates and four *Fusarium* isolates were obtained, as shown in Table 2. *Trichoderma* colonies were identified based on the overall morphological characteristics of *Trichoderma* spp. To conduct further characterization, molecular identification, antagonism testing, and volatile organic compound (VOC) production studies, four rapidly growing isolates of Trichoderma species (U-53, U-41, J-3, and U-27) and two isolates of Fusarium species (U-57 and J-20). Colony pigmentation was the primary criterion for colonies isolated from rhizosphere soil or roots. Most *Trichoderma* spp. were isolated from coffee root plants. The chosen *Trichoderma* isolates exhibited the genus’ typical rapid growth rates, ranging from 15 to 19 mm/day. The Fusarium isolates grew at significantly slower rates (5.86–7.86 mm/day). The average daily growth rate (mm/day) of *Trichoderma* isolates on PDA plates varied significantly at 27 °C. The selected isolates’ growth rates were: *T. harzianum* 404.1 (U-53) at 19 mm/day, *T. longibrachiatum* (U-41) at 16.6 mm/day, and *T. asperellum* (J-3) and *T. koningii* (U-27) at 15 mm/day.

**Table 2:** Colony, site, origin, and isolation code of *Trichoderma* and *Fusarium* from rhizosphere soil and root samples.

Colonies	Site	Origin	Avg. growth rate (mm\day)	Code
*Trichoderma* spp.				
Colony 1	Jabal Shada Al-A’la	Coffee tree roots	19	U-53*
Colony 2	Wadi Al-Ain in Jazan	Coffee tree roots	15	J-3*
Colony 3	Jabal Shada Al-A’la	Coffee tree roots	16.6	U-41*
Colony 4	Jabal Shada Al-A’la	Rhizosphere soil	15	U-27*
Colony 5	Jabal Shada Al-Asfal	Coffee tree roots	12	D-41
Colony 6	Jabal Shada Al-Asfal	Coffee tree roots	15	D-35
Colony 7	Wadi Al-Ain in Jazan	Coffee tree roots	13.75	U-17
Colony 8	Wadi Al-Ain in Jazan	Coffee tree roots	14.5	D-15
*Fusarium* spp.				
Colony 9	Jabal Shada Al-A’la	Coffee tree roots	5.86	U-57*
Colony 10	Wadi Al-Ain in Jazan	Coffee tree roots	7.57	J-20*
Colony 11	Wadi Al-Ain in Jazan	Coffee tree roots	5.01	D-5
Colony 12	Wadi Al-Ain in Jazan	Coffee tree roots	5.5	D-2

In contrast, the *Fusarium* isolates U-57 and J-20 demonstrated considerably slower growth rates. The isolate J-20 recorded the highest rate (7.86 mm/day), while isolate U-57 was the slowest (5.86 mm/day). These comparative measurements confirm a significant difference in growth rate between the two fungal genera.

Based on their superior growth performance, the fast-growing *Trichoderma isolates*, U-53, U-41, J-3, and U-27, were selected for further morphological characterization and molecular identification to determine their antifungal activity against pathogenic *Fusarium* isolates and to analyze their VOC compounds.

### 3.2 Characterization of isolates

Colonies from *Trichoderma* isolates were investigated microscopically. The colonies of all isolates were white at the beginning, with abundant mycelium. At 48 h, except for the J-3 isolate, U-53, J-3, and U-41 were green due to sporulation of the asexual structure’s conidia. Complete mycelial growth was obtained after 4 days of incubation. On the seventh day, different colors and morphologies of the produced colonies were observed across the four *Trichoderma* isolates.

**Figure 1:**
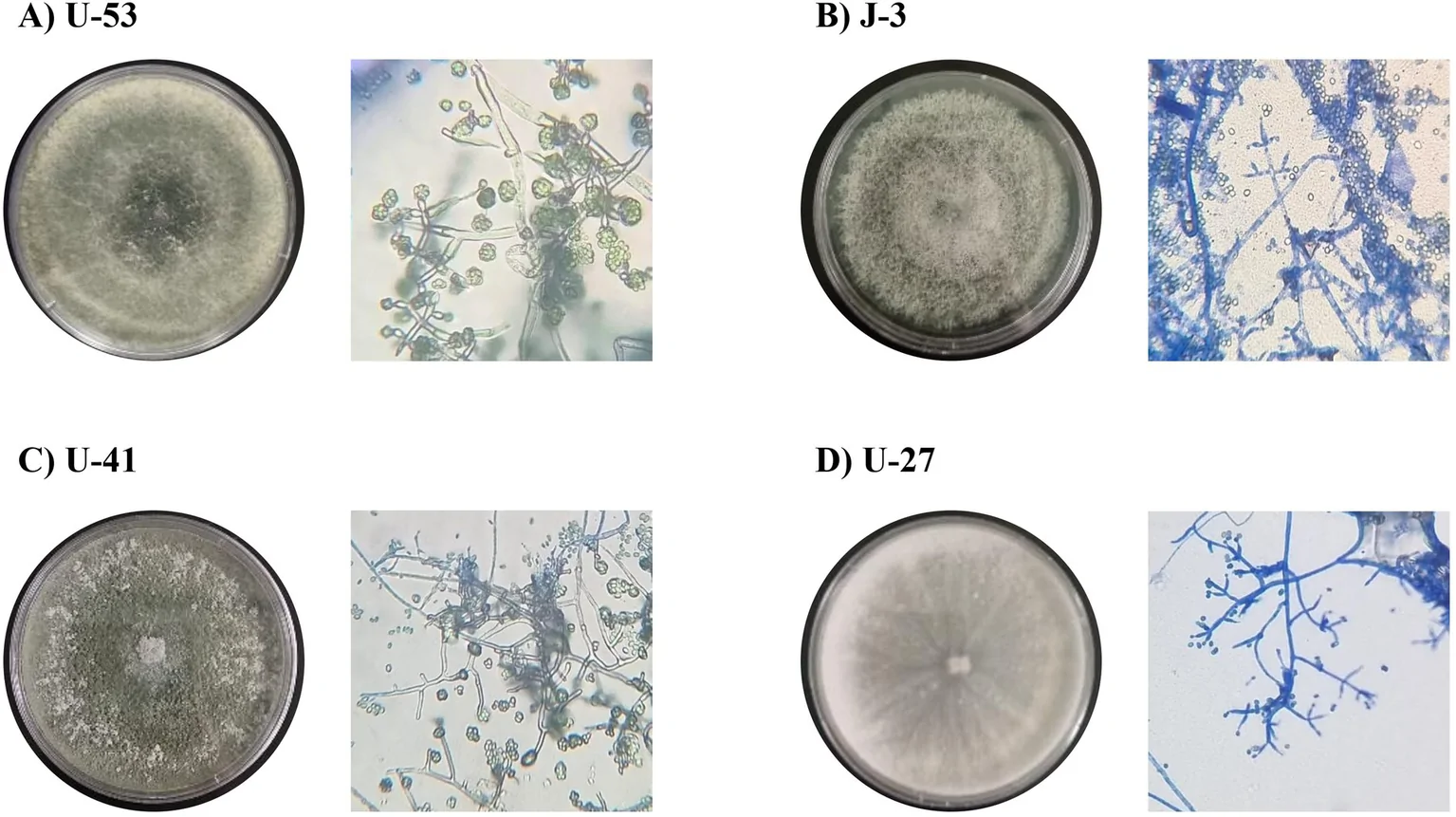
Mycelial growth of different *Trichoderma* isolates on PDA medium. The morphological characteristics were investigated microscopically (at 40X) after 7 days of incubation at 27 °C. **(A)** (U-53) *T. harzianum* 404.1, **(B)** (J-3) *T. asperellum*, **(C)** (U-41) *T. longibrachiatum*, **(D)** (U-27) *T. koningii*.

*Trichoderma* colonies (isolate U-53) were green with white edges; the back of the colony was light yellow, with a soft, cottony texture. The conidiophores were broad, verticillate, and frequently highly branching at 90°. The conidia were sub-globose with rough walls. This isolate was identified as *T. harzianum* (Figure 1A).

*Trichoderma* colonies (isolate J-3) were green, interlaced with white, with dark green edges, and the bottom of the colony was colorless, with a woolly texture. Most of the conidiophores formed symmetrically paired along the main branches and axis. The branching patterns of the conidiophores were extensive, exhibiting a verticillate arrangement close to 90 degrees. Conidia were globose to obovoid and green under a light microscope. It was identified as *T. asperellum* (Figure 1B).

*Trichoderma* (isolate U-41) initially developed as white, fluffy colonies that subsequently transitioned to dark green. This change was accompanied by the emergence of white or light-yellow pustules, frequently arranged in concentric patterns, along with a diffusion of yellow pigment from the mycelium in the lower part of the PDA plate. The isolate produced branched conidiophores that were either straight or curved with short lateral branches. The conidia were generally ellipsoidal to oblong with a smooth texture. This isolate was identified as *T. longibrachiatum* (Figure 1C).

*Trichoderma* colonies (isolate U27) were white, soft, and cottony. It was gradually turning pale green; however, the base of the colony remained white. The primary and secondary branches of conidiophores arose at a right-angle and branched. They were organized in a pyramidal structure with longer branches at the base and progressively shorter branches near the tip. Phialides were cylindrical to sharply constricted at the tips. This isolate produced smooth-walled, egg-shaped conidia from the ends of the phialides. The isolate was identified as *T. koningii* (Figure 1D).

**Figure 2:**
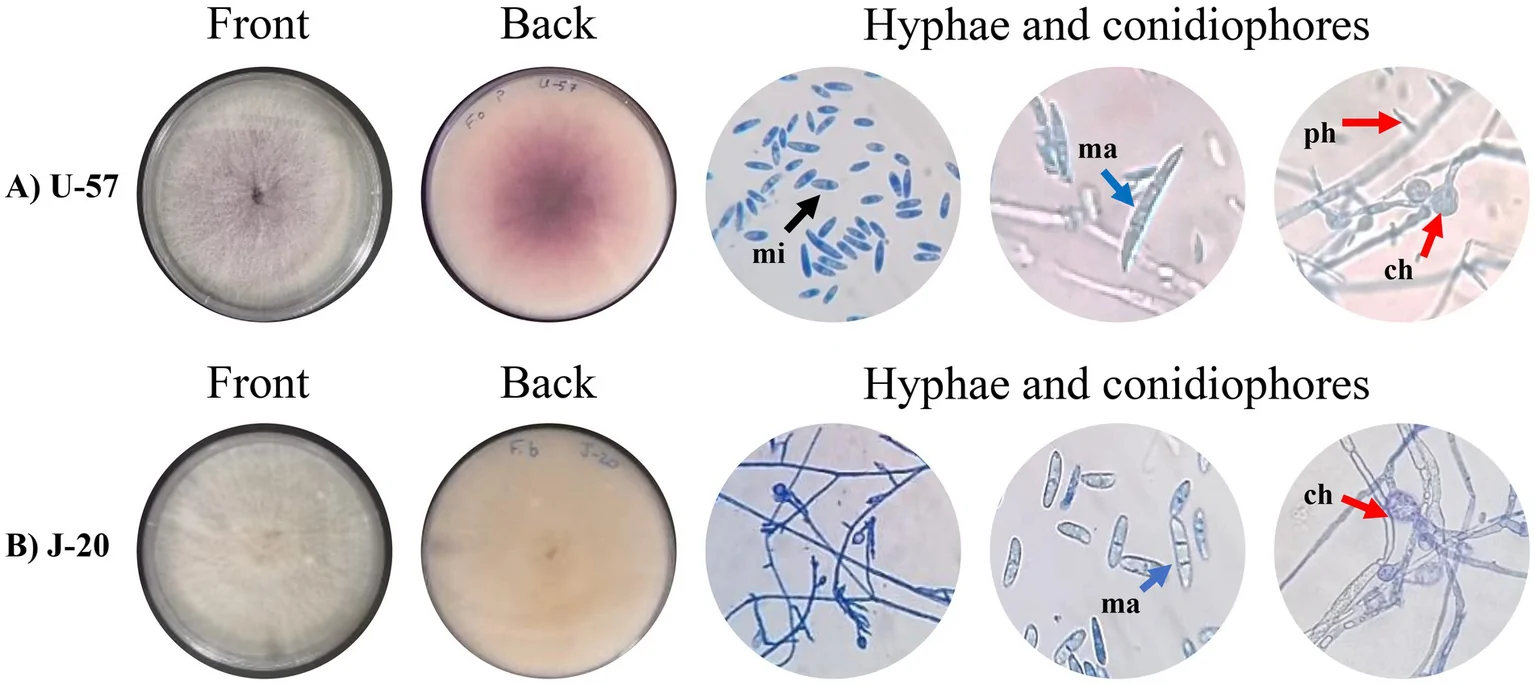
Mycelial growth of different *Fusarium* isolates on PDA medium. The morphological characteristics were investigated microscopically (at 40X and 100X). **(A)** (U-57) *F. oxysporum*: Colony morphology (front and reverse views), hyphae and conidiophores (black arrow), abundant microconidia, macroconidia (blue arrow), and phialides (ph) and chlamydospores (ch) (red arrows), **(B)** (J-20) *F. brachygibbosum*: Colony morphology (front and reverse views), conidiophores and hyphae, macroconidia (ma) (blue arrow), and chlamydospores (ch) (red arrow).

Morphological identification of the recovered fungal isolates was performed based on colony characteristics on PDA and microscopic examination of conidial structures after incubation at 27 °C for 5–7 days. The isolates were identified as *F. oxysporum* (U-57) and *F. brachygibbosum* (J-20) based on their cultural and microscopic characteristics (Figure 2).

Isolate (U-57) produced abundant white cottony aerial mycelium that gradually developed pale purple pigmentation on PDA. The reverse side also showed pale purple pigmentation. Microscopic observations revealed abundant oval-to-reniform microconidia, mostly aseptate, typically formed in false heads on short monophialides, whereas macroconidia were falcate with 3–4 septa. The chlamydospores were spherical to subspherical, with smooth and thick walls. They formed terminally or intercalary in the hyphae, singly, in pairs, or in short chains (Figure 2A). This strain was identified as *F. oxysporum*.

*Fusarium* (isolate J-20) formed dense cottony colonies that were initially white, with light brown coloration on the reverse side of the plate. Microscopic examination revealed oval to ellipsoidal microconidia and moderately curved macroconidia with 3–5 septa. The chlamydospores were abundant and considered a characteristic feature. They were spherical, smooth-walled to slightly rough, often thick-walled, and formed intercalary or terminally in chains of 2–3 cells (Figure 2B). These strains were identified as *Fusarium brachygibbosum*.

### 3.3 Identification of *Trichoderma* and *Fusarium* spp.

Molecular identification of the *Trichoderma* isolates U-53, J-3, U-41, and U-27 by ITS1 and ITS4 showed that they were *T. harzianum* 404.1, *T. asperellum*, *T. longibrachiatum,* and *T. koningii*, respectively. The analyses also showed that the isolates U-57 and J-20 were *F. oxysporum* and *F. brachygibbosum,* respectively. The names were supported by morphological data.

**Figure 3:**
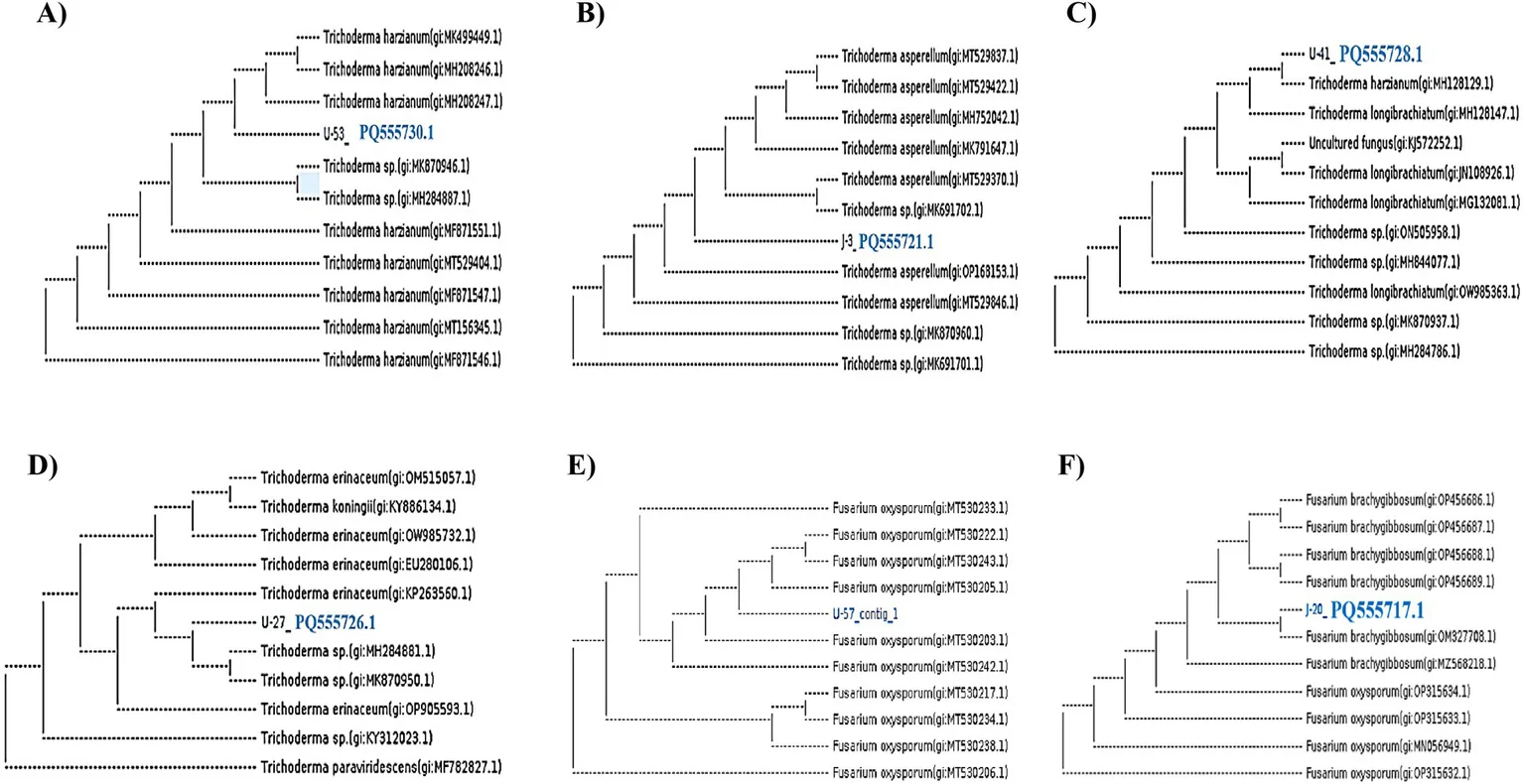
Phylogenetic analysis of 18S rDNA of the isolates with accession numbers in NCBI GenBank. **(A)** Isolate (U-53) *T. harzianum* 404.1 (PQ555730.1), **(B)** isolate (J-3) *T. asperellum* (PQ555721.1), **(C)** isolate (U-41) *T. longibrachiatum* (PQ555728.1), and **(D)** isolate (U-27) *T. koningii* (PQ555726.1). **(E)** isolate (U-57) *F. oxysporum* (PQ555706.1), and **(F)** isolate (J-20) *F. brachygibbosum* (PQ555717.1).

The analysis of 18S rDNA of the *Trichoderma* isolates, by amplifying the (ITS) region with primers ITS1 and ITS4 and the BlastN search, revealed 99–100% similarity with 0.0 E-Value to *T. harzianum* 404.1 (PQ555730.1), *T. asperellum* (PQ555721.1), *T. longibrachiatum* (PQ555728.1), and *T. koningii* (PQ555726.1) in NCBI GenBank, as shown in Figures 3A–D.

For *Fusarium* isolates, molecular identification was also performed by amplifying and sequencing the ITS region using the universal primers ITS1 and ITS4. The obtained sequences were compared with those available in GenBank using BLAST analysis. The isolates showed high sequence similarity with reference sequences of *F. oxysporum* and *F. brachygibbosum*, supporting their identification in combination with morphological characteristics.

The accession numbers for the *Fusarium* isolates were as follows: (U-57) *F. oxysporum* (PQ555706.1) and (J-20) *F. brachygibbosum* (PQ555717.1), as previously registered in NCBI GenBank on 04 November 2024 (Figures 3E,F).

### 3.4 *In vitro* antagonistic activity of *Trichoderma* isolates against *F. oxysporum* and *F. brachygibbosum*

**Figure 4:**
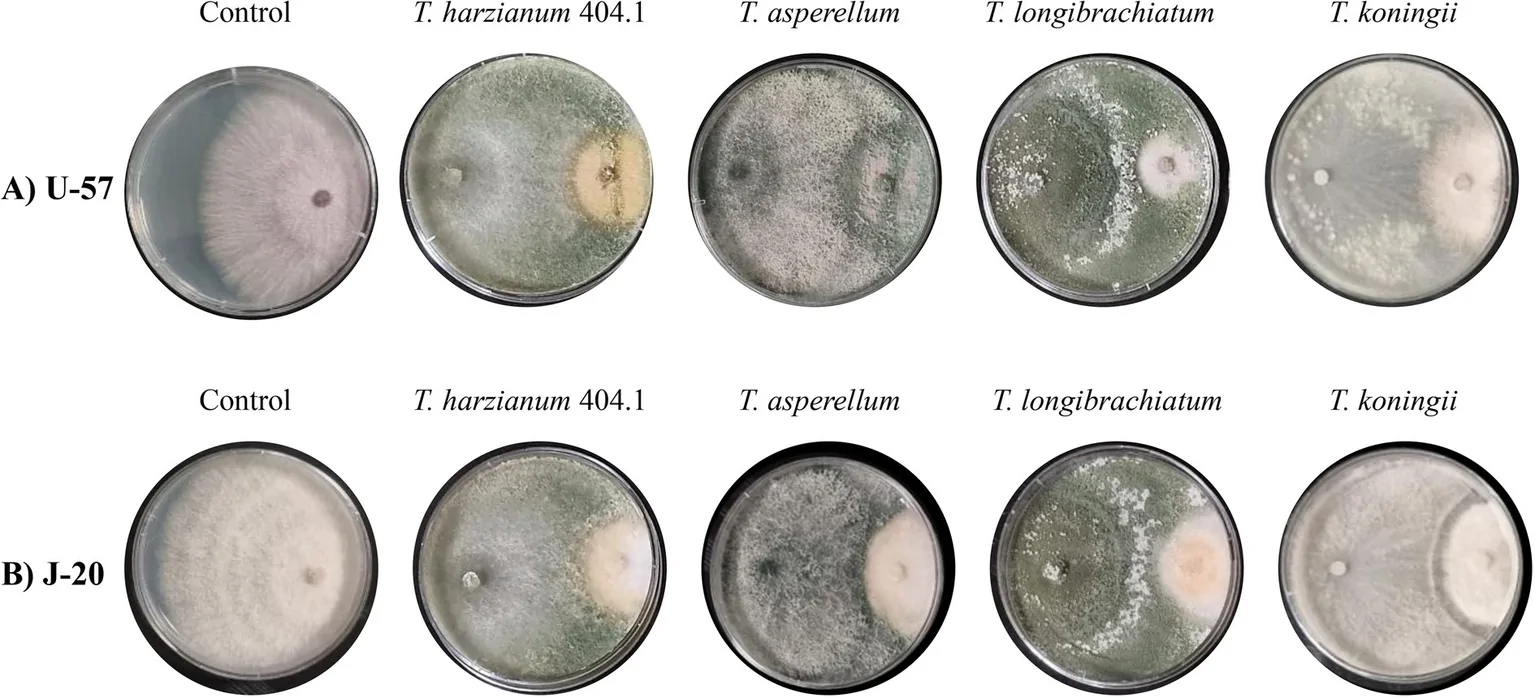
Dual culture assay showing in vitro antagonistic activity of *Trichoderma* spp. against *Fusarium* isolates on PDA at day 7. **(A)** *F. oxysporum* U-57 as control, *T. harzianum* 404.1*, T. asperellum*, *T. longibrachiatum*, an*d T. koningii*, respectively, against *F. oxysporum* U-57. **(B)** *F. brachygibbosum* J-20 as control, *T. harzianum* 404.1*, T. asperellum*, *T. longibrachiatum*, an*d T. koningii*, respectively, against *F. brachygibbosum* J-20.

The antifungal activity of the four *Trichoderma* isolates against the two *Fusarium* strains was evaluated in the dual culture experiment (Figure 4). All *Trichoderma* isolates exhibited inhibitory activity starting from day 3 of incubation. The average inhibition rate ranged from 65.33 to 77.60%.

*Fusarium* growth was inhibited as compared to the control. *Fusarium oxysporum* (U-57) colony growth decreased from 50 mm to 17.33 mm with *T. koningii* (U-27), while the decrease was greater with *T. longibrachiatum* (U-41), ranging from 50 mm to 12 mm. However, when antagonized with *F. brachygibbosum* (J-20), mycelial growth decreased from 65 mm to 17.67 with *T. longibrachiatum* (U-41), while the growth decrease was even greater when confronted with *T. harzianum* 404.1 (U-53) and *T. asperellum* (J-3) (Table 3).

**Table 3:** Effect of *Trichoderma* spp. on the mycelial growth of different *Fusarium* pathogen species.

Pathogens	Species of *Trichoderma*	The mean diameter of mycelial growth of phytopathogens (mm)				
		Control	7th day	% growth	t	*P*-value
*F. oxysporum* (U-57)	*T. harzianum* 404.1 (U-53)	50 ± 2	13.17 ± 0.29	26.33	28.3	0.001**
	*T. asperellum* (J-3)		14.67 ± 1.53	29.33	19.04	0.003*
	*T. longibrachiatum* (U-41)		12 ± 1	24	38	0.001**
	*T. koningii* (U-27)		17.33 ± 1.53	34.67	19.6	0.003*
*F. brachygibbsum* (J-20)	*T. harzianum* 404.1 (U-53)	65 ± 1.15	14.83 ± 0.29	22.82	84.59	<0.001**
	*T. asperellum* (J-3)		14.67 ± 0.58	22.56	51	<0.001**
	*T. longibrachiatum* (U-41)		17.67 ± 2.31	27.18	41.57	0.001**
	*T. koningii* (U-27)		16.33 ± 1.53	25.13	41.05	0.001**

**Figure 5:**
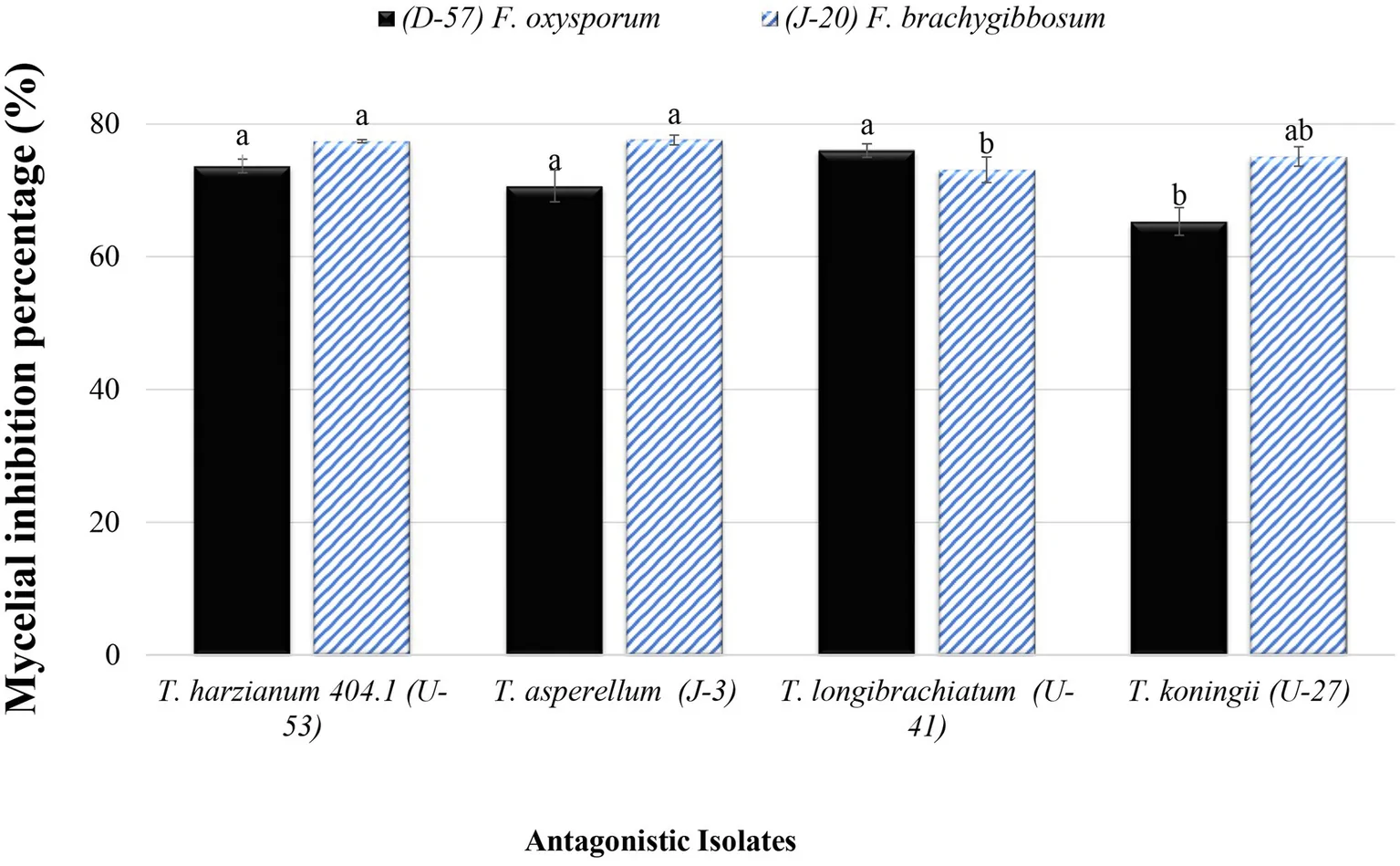
Antagonistic activity of *Trichoderma* isolates against *Fusarium* pathogens causing root disease of coffee plants.

The mycelial inhibition percentage of *Fusarium oxysporum* by the *Trichoderma* antagonistic strains was 73.67% by *T. harzianum* 404.1 (U-53), 70.67% by *T. asperellum* (J-3), 76% by *T. longibrachiatum* (U-41), and 65.33% by *T. koningii* (U-27). The inhibition percentage of *Fusarium brachygibbosum* by *Trichoderma* strains was *T. harzianum* 404.1(U-53) 77.40%, *T. asperellum* (J-3) 77.60%, *T. longibrachiatum* (U-41) 73.10, and 75.10% *T. koningii* (U-27) (Table 4; Figure 5). A microscopic examination of the sites of interaction between strains of fungal pathogens and antagonists provided data on mycoparasitism by various *Trichoderma* spp. In confrontation experiments between *F. brachygibbosum* and *T. asperellum* (J-3), *T. asperellum* displayed the highest mycoparasitism score (3.8 ± 0.4) of all antagonists tested on this pathogen (Table 3). As estimated by the number of sites of hyphal coiling and associated penetration structures, mycoparasitism by this isolate was extensive (coiling of hyphae of the pathogen in more than 50% of the microscopic fields examined). Also, considerable mycoparasitism (score 3.2 ± 0.3) by *T. harzianum* 404.1 (U-53) on both *F. brachygibbosum* and *F. oxysporum* was found. Coiling of hyphae and associated hyphal swelling were observed in 25–50% of the fields examined. In contrast, mycoparasitism by the other two species tested, *T. longibrachiatum* (U-41) and *T. koningii* (−27), was predominantly of a contact type and low (score 1.8–2.4). No penetration structures were found. The degree of mycoparasitism exhibited by the various antagonists tested on the two fusaria correlated with their ability to inhibit growth of the pathogen tested (r = 0.78, *p* = 0.02). Thus, it appears that physical mycoparasitism can be a major factor in the ability of *Trichoderma* spp. to interact with and inhibit the growth of various fungal pathogens. However, it is also clear that additional factors must be involved, since the degree of inhibition of growth of various fusaria by the antagonists tested was not entirely accounted for by their operatively interactive behavior.

**Table 4:** *In vitro* mycelial inhibition (%) of *Fusarium* isolates by *Trichoderma* spp. on day 7.

Pathogens	*Trichoderma* isolates	Inhibition (%)	Mycoparasitism score (0–4)	Dominant interaction type
*F. oxysporum* (U-57)	*T. harzianum* 404.1 (U-53)	73.67 ± 1.02 ^a^	3.1 ± 0.3	Coiling + swelling
	*T. asperellum* (J-3)	70.67 ± 2.4 ^a^	3.2 ± 0.4	Coiling + penetration
	*T. longibrachiatum* (U-41)	76.00 ± 1 ^a^	2.4 ± 0.3	Contact only
	*T. koningii* (U-27)	65.33 ± 2.08 ^b^	1.8 ± 0.4	Limited contact
*F. brachygibbosum* (J-20)	*T. harzianum* 404.1 (U-53)	77.40 ± 0.25 ^a^	3.3 ± 0.2	Coiling + swelling
	*T. asperellum* (J-3)	77.60 ± 0.74 ^a^	3.8 ± 0.4	Extensive coiling + penetration
	*T. longibrachiatum* (U-41)	73.10 ± 1.94 ^b^	2.1 ± 0.2	Contact only
	*T. koningii* (U-27)	75.10 ± 1.46 ^ab^	2.2 ± 0.3	Contact only

Values are presented as mean ± SE (*n* = 3). Means followed by the same letter within each column are not significantly different according to Duncan’s multiple range test at *P* ≤ 0.05.

The Pearson correlation coefficient between mycoparasitism and inhibition was significant (r = 0.78, *p* = 0.02), but this relationship did not remain significant in the multiple regression model (*p* = 0.18). The reason behind this discrepancy is the fact that the mycoparasitism scores are associated with growth rate; *Trichoderma* isolates with faster growth rates had larger mycoparasitism scores. Once the growth rate is controlled in the model, the mycoparasitism explains very little additional variance in the inhibition, indicating that the relationship between mycoparasitism and inhibition is mostly due to the shared variance with the growth rate and not the mycoparasitic effect itself.

### 3.5 GC–MS analysis of *Trichoderma* spp.

**Figure 6:**
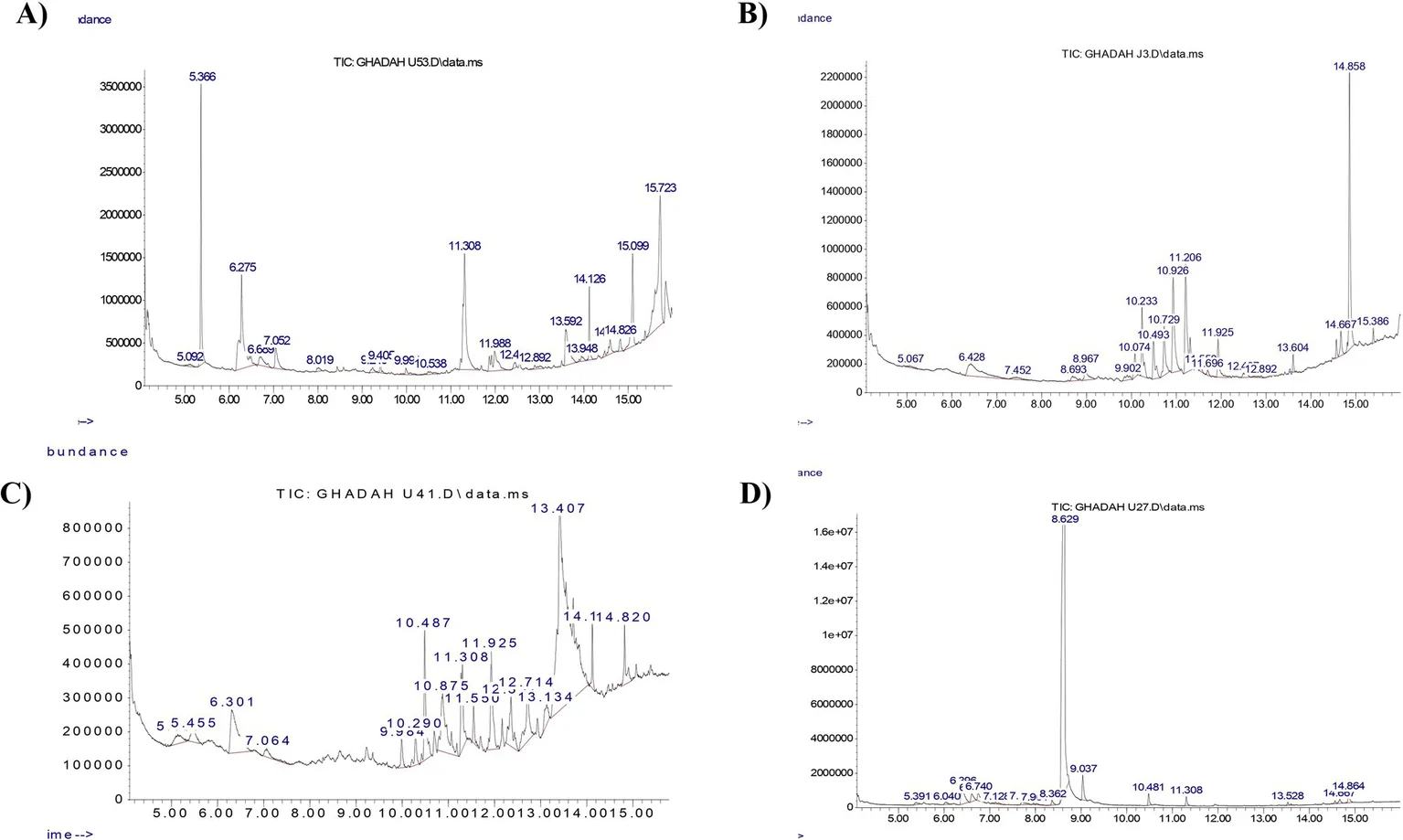
Gas chromatography–mass spectroscopy (GC–MS) of crude ethyl acetate extracts from *Trichoderma* species. **(A)** *T. harzianum* 404.1 U-53, **(B)** *T. asperellum* J-3, **(C)** *T. longibrachiatum* U-41, **(D)** *T. koningii* U-27. Peaks representing recognized volatile organic compounds are labeled with respective retention durations. Each extract’s total ion chromatogram (TIC) is displayed.

The GC–MS analysis of ethyl acetate crude extracts from four *Trichoderma* spp. revealed a diverse array of VOCs and extracellular metabolites, which are listed in Table 5. Twenty VOCs were identified from *T. harzianum* 404.1 (U-53), 18 from *T. asperellum* (J-3), 15 from *T. longibrachiatum* (U-41), and 16 from *T. koningii* (U-27). Typical chromatograms and mass spectra of the compounds identified from the ethyl acetate extracts of *Trichoderma* isolates are shown in Figure 6.

**Table 5:** VOCs identified by GC–MS.

Sn	RT (min)	Area (%)	Absolute height (Ab)	Name of the compound	Mol weight (amu)
*T. harzianum* 404.1 (U-53)					
1	5.366	0.46	3,695,629	Eucalyptol	154.136
2	6.275	11.88	1,299,407	Phenylethyl Alcohol	122.073
3	6.689	14.74	340,318	Ethanol, 2-(2-ethoxyethoxy)-, acetate	176.105
4	7.052	2.06	441,252	Acetic acid, cyclohexyl ester	142.099
5	8.019	2.70	213,275	2-Butanone, 4-(acetyloxy)-	130.063
6	9.24	0.52	213,542	7-Oxabicyclo[4.1.0]heptane, 1-methyl-4-(2-methyloxiranyl)-	168.115
7	9.405	0.59	264,314	1-Naphthalenol, decahydro-1,4a-dimethyl-7-(1-methylethylidene)-, [1R-(1.alpha.,4a.beta.,8a.alpha.)]-	222.198
8	9.991	0.624	206,534	1H-Benzocyclohepten-7-ol, 2,3,4,4a,5,6,7,8-octahydro-1,1,4a,7-tetramethyl-, cis-	222.198
9	10.538	0.96	167,005	. beta.-l-Rhamnofuranoside, methyl-5-O-acetyl-	220.095
10	11.308	0.83	1,547,844	1,2-Benzenedicarboxylic acid, butyl 2-ethylhexyl ester	334.214
11	11.988	15.95	406,020	3,8-Dioxatricyclo [5.1.0.0(2,4)] octane, 4-ethenyl-	138.068
12	12.453	5.28	276,363	Benzenemethanol, alpha.,.alpha.,4-trimethyl-	150.104
13	12.892	0.59	242,737	Spiro [4.5]decan-7-one, 1,8-dimethyl-8,9-epoxy-4-isopropyl-	236.178
14	13.592	0.83	663,631	1-Hydroxy-4-methylanthraquinone	238.063
15	13.948	6.12	347,244	Cyclohexane, (1-octylnonyl)-	322.36
16	14.126	0.71	1,160,253	Hexanedioic acid, bis(2-ethylhexyl) ester	370.308
17	14.59	3.15	540,043	Ledene oxide-(II)	220.183
18	14.826	1.92	562,578	3-n-Butylthiolane, S, S-dioxide	176.087
19	15.099	1.02	1,548,057	4,22-Stigmastadiene-3-one	410.355
20	15.723	7.13	2,225,679	2,6-Lutidine, 3,5-dichloro-4-cyclohexylthio-	289.046
*T. asperellum* (J-3)					
1	8.693	1.50	116,153	Methyl 2-methylsulfonyl-.alpha.-d-xylofuranoside	242.046
2	8.967	2.89	189,683	Ethanone, 2-hydroxy-1,2-diphenyl-	212.084
3	9.902	1.54	123,588	Undeca-3,4-diene-2,10-dione, 5,6,6-trimethyl-	222.162
4	10.074	2.77	271,648	Caryophyllene oxide	220.183
5	10.233	7.57	593,005	Cyclohexanemethanol, 4-ethenyl-.alpha.,.alpha.,4-trimethyl-3-(1-methylethenyl)-, [1R-(1.alpha.,3.alpha.,4.beta.)]-	222.198
6	10.493	5.47	355,773	Hydroxy-.alpha.-terpenyl acetate	212.141
7	10.729	6.70	462,200	trans-3-Caren-2-ol	152.12
8	10.926	13.88	803,887	Bicyclo[3.1.0]hex-2-ene, 2-methyl-5-(1-methylethyl)-	136.125
9	11.206	17.25	895,683	2-Furanmethanol, tetrahydro-.alpha.,.alpha.,5-trimethyl-5-(4-methyl-3-cyclohexen-1-yl)-, [2S-[2.alpha.,5.beta.(R*)]]-	238.193
10	11.55	0.66	186,629	trans,trans-2,6-Dimethyl-2,6-octadiene-1,8-diol	170.131
11	11.696	0.57	157,978	2,6,10,14-Pentadecanetetrone	268.167
12	11.925	5.09	375,454	1,2-Benzenedicarboxylic acid, butyl 2-methylpropyl ester	278.152
13	12.497	1.29	139,488	Cyclohexanol, 2-methyl-3-(1-methylethenyl)-, acetate, (1.alpha.,2.alpha.,3.alpha.)-	196.146
14	12.892	0.61	118,651	2,5-Diacetyl-terephthalic acid	250.048
15	13.604	1.20	266,839	Andrographolide	350.209
16	14.667	3.84	429,441	n-Octadecanoic acid, dimethyl(trimethylsilylmethyl)silyl ester	428.351
17	14.858	26.20	2,313,752	Methanol, 6,8,9-trimethyl-4-(2-phenylethyl)-3-oxabicyclo[3.3.1]non-6-en-1-yl)-	300.209
18	15.386	0.87	450,395	2,3,4,6-Tetrafluorophenyl isothiocyanate	206.977
*T. koningii* (U-27)					
1	5.391	0.59	294,419	1-Fluorooctane	132.131
2	6.04	0.57	307,444	1-Nonyne	124.125
3	6.396	5.21	1,217,730	Cyclohexane, 2-propenyl-	124.125
4	6.606	1.71	800,613	3-Cyclopentyl-1-propanol	128.12
5	6.74	1.80	868,733	11-Dodecen-1-yl acetate	226.193
6	7.128	0.65	303,368	2-Decenal, (E)-	154.136
7	7.707	1.01	326,458	Bicyclo[2.2.1]heptan-2-ol, acetate	154.099
8	7.961	0.51	259,366	l-Gala-l-ido-octose	240.085
9	8.362	0.79	440,508	2-Dimethylamino-6,7-dihydroimidazo[1,2-a][1,3,5]triazin-4(8H)-one	181.096
10	8.629	78.98	17,080,250	2H-Pyran-2-one, 6-pentyl-	166.099
11	9.037	3.03	1,888,478	Phenol, 3-ethyl-	122.073
12	10.481	1.26	825,182	Bicyclo[2.2.2]octane, 1,2,3,6-tetramethyl-	166.172
13	11.308	1.01	643,572	1,2-Benzenedicarboxylic acid, butyl 2-ethylhexyl ester	334.214
14	13.528	0.40	323,130	9,19-Cyclolanostan-3-ol, acetate, (3.beta.)-	470.412
15	14.667	1.02	488,776	Medroxyprogesterone Acetate	386.246
16	14.864	1.46	888,768	Methanol, 6,8,9-trimethyl-4-(2-phenylethyl)-3-oxabicyclo[3.3.1]non-6-en-1-yl)-	300.209
*T. longibrachiatum* (U-41)					
1	6.301	7.19	265,846	Phenylethyl Alcohol	122.073
2	7.064	1.09	151,369	Ethyl Acetate	88.052
3	9.984	1.21	178,097	1H-Benzocyclohepten-7-ol, 2,3,4,4a,5,6,7,8-octahydro-1,1,4a,7-tetramethyl-, cis-	222.198
4	10.29	1.53	202,455	2,6,10-Dodecatrien-1-al, 12-(acetoxy)-2,6,10-trimethyl-, (E, E, E)-	278.188
5	10.487	5.72	498,973	8-Dodecen-1-ol, acetate, (Z)-	226.193
6	10.875	7.48	312,275	4-(2,6,6-Trimethyl-cyclohexa-1,3-dienyl)-pent-3-en-2-one	204.151
7	11.308	4.57	397,951	1,2-Benzenedicarboxylic acid, butyl octyl ester	334.214
8	11.55	1.15	276,279	3,8-Nonadien-2-one, (E)-	138.104
9	11.925	5.43	438,537	1-Methyl-1-hydroxymethyladamantane	180.151
10	12.357	3.76	303,151	Benzazepine, 1,2,3,4-tetrahydro-N-acetyl-7,8-dimethoxy-1-methylene-	261.136
11	12.714	4.59	323,826	Adamantane	136.125
12	13.134	1.72	280,166	Artemiseole	152.12
13	13.407	50.64	877,704	9,10-Anthracenedione, 2,7-dimethyl-	236.084
14	14.12	1.4	517,834	Hexanedioic acid, bis(2-ethylhexyl) ester	370.308
15	14.82	2.44	515,119	Isoxazole, 4-(4-fluorobenzoyl)-5-methyl-3-phenyl-	281.085

The four *Trichoderma* spp. produced high levels of volatile organic compounds. The VOCs retention times ranged from 5.366 to 15.723 min. 2,6-Lutidine, 3,5-dichloro-4-cyclohexylthio- had the longest retention time, while Eucalyptol had the lowest.

Based on these results, some shared overlap of VOCs between the four *Trichoderma* isolates was identified as follows: (Phenylethyl Alcohol), (Hexanedioic acid, bis(2-ethylhexyl) ester), and (1H-Benzocyclohepten-7-ol, 2,3,4,4a,5,6,7,8-octahydro-1,1,4a,7-tetramethyl-, cis-) were found in *T. longibrachiatum* (U-41) and *T. harzianum* 404.1 (U-53). 1,2-Benzenedicarboxylic acid, butyl 2-ethylhexyl ester was found in *T. harzianum* 404.1 (U-53) and *T. koningii* (U-27). Methanol, 6,8,9-trimethyl-4-(2-phenylethyl)-3-oxabicyclo.3.3.1] non-6-en-1-yl)- was found in *T. koningii* (U-27) and *T. asperellum* (J-3).

Out of the 69 VOCs identified, 12 of them have been reported to possess antifungal activities (Zou et al., 2022; Wu et al., 2023; Liao et al., 2024; Singh et al., 2024). The relative abundance of the mechanics-linked VOCs was significantly different between *Trichoderma* spp. (*F* = 8.34, *p* = 0.003). The highest percentage of mechanism-linked VOCs was observed in *T. asperellum* JJ3, with a cumulative percentage of 42.3% of the total peak area. The major mechanism-linked VOCs in *T. asperellum* J-3 were caryophyllene oxide (18.7%) and *α*-thujene (12.4%). *T. harzianum* 404.1 had 38.6% of mechanism-linked VOCs, with 1,8-cineole (15.52%) and phenylethyl alcohol (11.8%) being the major compounds. Pearson’s correlation was calculated between the relative growth of the pathogen and the percentage of the peak area for individual VOCs in both fungi. For the inhibition of *F. brachygibbosum*, the highest positive correlation was to caryophyllene oxide (r = 0.911, *p* = 0.003), followed by 1,8-cineole (r = 0.76, *p* = 0.04) and 2-phenylethanol (r = 0.72, *p* = 0.05). In contrast, the growth of *F. oxysporum* was most strongly correlated to 6-pentyl-2H-pyran-2-one (r = 0.85, *p* = 0.01). Notably, this VOC is only produced by *T. koningii* U-27.

Stepwise multiple regression, results provided in Table 6, revealed that the *Trichoderma* growth rate alone explained 52.3% of the variance in *Fusarium* inhibition (adjusted R^2^ = 0.523; *F* = 8.76; df = 1, 16; *p* = 0.02). The inclusion of the ‘number of mechanism-linked VOCs’ increased the explained variance to 73.8% (ΔR^2^ = 0.215; *p* = 0.01). The results revealed that the caryophyllene oxide emerged as the single strongest predictor for *F. brachygibbosum* inhibition (*β* = 0.68; *p* = 0.003), whereas the 6-pentyl-2H-pyran-2-one emerged as the strongest predictor for *F. oxysporum* inhibition (*β* = 0.59; *p* = 0.008). The Mycoparasitism score did not enter the final model (*p* = 0.18), suggesting that in this *in vitro* system, the VOC-mediated mechanisms and growth rate competition are more significant contributors to *Fusarium* inhibition than physical mycoparasitism.

**Table 6:** Hierarchical regression analysis splitting the variance in *Fusarium* inhibition among *Trichoderma* pathways.

Model	Predictor variable	β-coefficient ±SE	t	ΔR^2^	ΔF	*p-*value
*F. brachygibbosum*	*Trichoderma* growth rate	0.52 ± 0.18	2.96	0.523	8.76	0.02
	Mechanism-linked VOC count	0.38 ± 0.12	3.21	0.215	6.54	0.01
	Caryophyllene oxide (presence)	0.68 ± 0.15	4.53	0.112	12.32	0.003
*F. oxysporum*	*Trichoderma* growth rate	0.48 ± 0.19	2.53	0.498	7.94	0.03
	Mechanism-linked VOC count	0.42 ± 0.14	3	0.198	5.21	0.01
	6-PP (presence)	0.59 ± 0.17	3.47	0.089	3.33	0.008

ΔR^2^ shows the change in explained variance by the addition of each set of variables; ΔF: change in F value for the added variables (ΔF = (ΔR^2^ / df₁)/((1 - R^2^_full)/df₂), with ΔR^2^ = increase in R^2^ after adding new variable(s), df₁ = number of variables added (degrees of freedom for numerator), R^2^_full = R^2^ f full model (after all variables added), df₂ = number of samples - number of parameters - 1); all final models were significant at *p* < 0.01. Note that Mechanism-linked VOCs refers to specific VOCs that interact with antifungal molecular targets, which have been previously reported (1,8-cineole, caryophyllene oxide, 6-pentyl-2H-pyran-2-one, 2-phenylethanol).

### 3.6 Hypothesis testing summary

Three hypotheses proposed in the introduction have been tested in the following way:

- The strains *T. harzianum* 404.1, *T. asperellum* J-3, *T. longibrachiatum* U-41, and *T. koningii* U-27 produce 20, 18, 15, and 16 compounds, respectively, based on the results of GC–MS analysis (H₁). Three VOCs (phenylethyl alcohol, hexanedioic acid bis(2-ethylhexyl) ester, and 1H-benzocyclohepten-7-ol derivative) are common to all four species, which proves species-specific VOC fingerprinting (χ^2^ = 34.2, df = 9, *p* < 0.001).
- *Trichoderma* growth rate was a major factor that accounted for 52.3% of the variance in the inhibition of *Fusarium* (adjusted R^2^ = 0.523; F = 8.76; *p* = 0.02). When the mechanism-linked VOC count was added to the model, the explained variance increased by 73.8% (ΔR^2^ = 0.215; *p* = 0.01). The growth rate is the main predictor of inhibition; however, VOCs play an important role as well (ΔF = 6.54; *p* = 0.01).
- The caryophyllene oxide compound was formed only by *T. asperellum* J-3 and showed the most significant relationship with inhibition of *F. brachygibbosum* growth (r = 0.911; *p* = 0.003). The 6-pentyl-2H-pyran-2-one compound was isolated from *T. koningii* U-27 alone, and this compound showed the greatest relationship with inhibition of *F. oxysporum* growth (r = 0.85, *p* = 0.01). These data indicate the species-specific distribution of VOCs associated with distinct mechanisms.

## 4 Discussion

The most important features of the *Trichoderma* colonies isolated from coffee roots and the rhizosphere were identified using culture and morphological characteristics. These species were found more often in the roots of coffee plants than in the surrounding soil. This suggests that *Trichoderma* can effectively colonize coffee plant roots (Pozo et al., 2024). In this nonpathogenic, symbiotic relationship, *Trichoderma* taxa invade the plant’s root system and live within living tissues (Mulaw et al., 2013; Pozo et al., 2024). It has been observed that *Trichoderma* spp. grow faster than pathogenic taxa, as rapid growth is one of the most important characteristics of *Trichoderma*. In our study, *Trichoderma* spp. occupied a larger area in a Petri dish than *Fusarium* spp., indicating clear competition between them for both nutrients and space (Kumari et al., 2024; El-Dawy et al., 2025). Also, it pointed out that the faster radial growth of *Trichoderma* sp. compared to *Fusarium* sp. allowed the antagonistic organism to colonize the medium quickly and overcome the pathogen colony, which is a key feature of effective biological control. Nevertheless, the isolates demonstrated a degree of variability in their macroscopic characteristics, including colony morphology and mycelial attributes, as well as the rate of colony expansion. Furthermore, they also exhibited differences in microscopic features, such as the characteristics of conidia and phialides.

In addition to identifying species that inhibit *Fusarium*, this study also assessed the contribution of three different mechanisms or layers of action to the inhibition of *Fusarium* by *Trichoderma* spp. in coffee. Our data provided the first quantitative estimate of the amount of variance in the inhibition of *Fusarium* by *Trichoderma* spp. explained by the growth rate of the colonizing *Trichoderma* spp. (52%). This result supports the competitive exclusion model of antagonism of fungal pathogens by *Trichoderma* spp. proposed by Harman et al. (2004). For VOCs, the amount of variance in the inhibition of *Fusarium* by *Trichoderma* spp. (2–25%) is linked to the three different mechanisms of action, in addition to that explained by the growth rate of the colonizing *Trichoderma* spp., and indicates that the VOCs involved in inhibition of *Fusarium* are not just additional to the competitive exclusion of pathogen colonization of the rhizosphere by *Trichoderma* spp. but are also synergistic with this mechanism of action. A larger amount of biomass of a *Trichoderma* spp. would not only allow for the occupation of a larger volume of space in the rhizosphere to exclude a pathogen from colonization of this niche, but would also provide a greater source for the emission of VOCs that are involved in the inhibition of *Fusarium* (Li et al., 2018). The amount of variance in the inhibition of *Fusarium* by *Trichoderma* spp. explained by mycoparasitism scoring was not significant when accounting for growth rate (*p* = 0.18), and therefore, in the confined environment of a Petri dish, contact-dependent mycoparasitism is apparently superseded by contact-independent mechanisms of inhibition of *Fusarium* by *Trichoderma* spp. It does not mean that physical mycoparasitism has no biological significance; on the contrary, the Pearson correlation coefficient between mycoparasitism and the inhibition was high (r = 0.78, *p* = 0.02). What it means is that in the small space of Petri dishes, the specific role of physical mycoparasitism in the process of inhibition is secondary to the impacts of the competition for growth rates and VOC inhibition because they share variance with mycoparasitism (the correlation between the latter two variables is 0.65). Therefore, under *in vitro* conditions, non-contact mechanisms are the main inhibitors, while mycoparasitism can be significant indirectly or in field conditions. This latter group of mechanisms of action would, however, be expected to be of even greater importance under field conditions, where the degree of separation of individual units of *Trichoderma* and *Fusarium* spp. would be greater than in a Petri dish, and therefore, the amount of inhibition of *Fusarium* by VOCs emitted by *Trichoderma* spp. would also be expected to be greater (Cook, 1993).

Culture and morphological traits used in identifying *Trichoderma* spp. are not enough to accurately tell the species apart. Therefore, molecular identification is important for confirming the species. Therefore, four isolates of *Trichoderma* and two isolates of *Fusarium* were identified to species level by using ITS sequences and morphology. Consequently, isolates of *T. harzianum* (PQ555730.1), *T. asperellum* (PQ555721.1), *T. longibrachiatum* (PQ555728.1), and *T. koningii* (PQ555726.1), in addition to *F. oxysporum* (PQ555706.1) and (J-20) *F. brachygibbosum* (PQ555717.1) were identified in this study. This result is consistent with del Carmen et al. (2021), who isolated several species of *Trichoderma* from the rhizosphere and root tissues of coffee plants. Mulatu et al. (2022) isolated *Trichoderma* spp., in addition to other species, from the rhizosphere soil of coffee, and *T. asperellum* was most abundant.

In the *in vitro* antagonistic activity of *Trichoderma* spp. against *F. oxysporum* and *F. brachygibbosum*, the observed inhibition percentages align well with published literature on *Trichoderma-Fusarium* interactions, which commonly report inhibition rates ranging from 50 to 90% depending on the isolate, pathogen, and experimental conditions (Rodríguez García and Wang Wong, 2020; Kumar et al., 2024; Browne et al., 2025). *T. longibrachiatum* (U-41) emerged as the most effective antagonist against *Fusarium oxysporum* with 76% inhibition. However, the same isolate showed relatively lower effectiveness (72.82%) against *F. brachygibbosum*. These percentages were higher than those obtained by Girma (2022), where the inhibition rate by this species reached 65.84% against the root pathogen *F. oxysporum* f. sp. *capsici* on hot pepper. However, in the study by El-Dawy et al. (2025), the inhibition activity of *T. longibrachiatum* ranged from 49.5–68.9% against *F. proliferatum.* This indicates that antagonistic capacity is not only species-dependent but also pathogen-specific. This differential effectiveness has been documented in multiple studies where *Trichoderma* isolates show varying performance against different target pathogens (Leslie and Summerell, 2006; Martínez-Coca et al., 2018; Rodríguez García and Wang Wong, 2020).

The performance reversal observed with *T. asperellum* J-3 (20 VOCs) and *T. harzianum* 404.1 U-53 (18 VOCs), showing moderate activity against *F. oxysporum* (70.67 and 73.67%, respectively) but highest activity against *F. brachygibbosum* (77.44 and 77.18%), underscores the importance of pathogen-specific screening. Despite having different VOC profiles, *T. harzianum* 404.1 and *T. asperellum* J-3 (18 VOCs) exhibited comparable levels of inhibition against *F. brachygibbosum*, which calls into question the notion that more VOC variety always translates into better biocontrol. Rather, our findings imply that the antifungal effectiveness of VOCs is determined by their particular composition rather than their overall quantity. Caryophyllene oxide (18.7% relative abundance) produced by *T. asperellum* J-3 had the highest association with the inhibition of *F. brachygibbosum* (r = 0.911, *p* = 0.003). On the other hand, 1,8-cineole (15.52%) and phenylethyl alcohol (11.8%) were generated by *T. harzianum* 404.1, and these compounds similarly demonstrated strong correlations (r = 0.76 and r = 0.72). This trend suggests that, if the molecule successfully targets the pathogen’s weaknesses, a single high-potency VOC can produce inhibition that is comparable to a more varied VOC profile. These results are consistent with research by Liao et al. (2024) and Singh et al. (2024), which showed that certain VOCs, like caryophyllene oxide and 1,8-cineole, have strong antifungal action against Fusarium species via different molecular mechanisms. Sehim et al. (2023) documented that *T. asperellum* strains exhibited variable antagonistic capacity against different *Fusarium* spp., with some isolates excelling against specific pathogens while showing moderate activity against others. Similarly, Kumari et al. (2024) found that an isolate of *T. harzianum* achieved approximately 72% inhibition against *Fusarium* wilt pathogens, comparable to a 73.67–77.18% range observed for *Trichoderma harzianum* 404.1 in this study. The isolate of *T. koningii* was characterized by low to moderate antagonistic activity only against *F. oxysporum* (65%), which is consistent with studies by Tkalenko et al. (2020), who determined the antagonistic activity of *T. koningii* against eight types of plant pathogenic fungi, including *F. oxysporum.* This highlights that species designation alone is insufficient for predicting biocontrol efficacy, and strain-level characterization is essential. In this work, *T. harzianum* 404.1 (U-53) and *T. asperellum* (J-3) were found to be the most effective, followed by other isolates. The rivalry for essential nutrients and spatial resources, mycoparasitism, as well as the production of antibiotic substances and hydrolytic enzymes, as articulated by Harman et al. (2004), constitute the fundamental mechanisms underpinning the antagonistic behavior exhibited by *Trichoderma.*

Notably, there were species-specific correlations between various VOCs and the inhibition of *F. brachygibbosum* and *F. oxysporum*. For example, the inhibition of *F. brachygibbosum* was strongly correlated with caryophyllene oxide, a compound recently shown to suppress ribosome synthesis in various *Fusarium* species by inhibiting lysine acetylation of histones (Liao et al., 2024). In contrast, the inhibition of *F. oxysporum* was strongly correlated with 6-pentyl-2H-pyran-2-one, which targets the TOR signaling pathway (Wu et al., 2023). This VOC can be produced solely by *T. koningii* U-27. As per the study conducted by Wu et al. (2023), where it was proven that 6-PP acts on the TOR signaling pathway in fungi, we may conclude that 6-PP from *T. koningii* acts in a similar way against *F. oxysporum*. Nonetheless, the present study lacks proof of 6-PP acting on the TOR pathway of *F. oxysporum,* and further research is required in this regard. This proves that there are mechanisms for resistance for each pathogen, and that *Trichoderma* spp. induces inhibition of TOR in *F. oxysporum* by means of 6-PP, while terpenoids like caryophyllene oxide affect *F. brachygibbosum* by other molecular targets. The species-specific susceptibility to these compounds could be the result of differences in cell wall composition or stress response pathways. *F. oxysporum* possesses a more robust oxidative stress response system compared to other *Fusarium* species (Singh et al., 2024), which could explain its lower susceptibility to caryophyllene oxide, which acts partly through the accumulation of reactive oxygen species (Liao et al., 2024). Alternatively, 6-PP was only detected in *T. koningii* cultures, and it exhibited the strongest correlation with the inhibition of *F. oxysporum* of all tested compounds. This compound and *T. koningii* may specifically target *F. oxysporum* and its various formae speciales, and further investigation of this hypothesis is warranted.

The analysis of the crude extracts from *Trichoderma* isolates by ethyl acetate extraction includes VOCs and extracellular antifungal compounds analyzed by GC–MS. The four species of *Trichoderma* differed in types and abundance of volatiles responsible for antifungal activity and many other compounds detected by this technique. All of these compounds identified were based on molecular weight, retention time, absolute height, and peak width. The main chemical groups of VOCs belonged to hydrocarbons, ketones, alkanes, alkenes, alkynes, alcohols, esters, aromatic compounds, ethers, oxides, sulfur compounds, and many polycyclic compounds (Guo et al., 2019; Elouark et al., 2025).

*T. harzianum* 404.1 (U-53) exhibited the most extensive metabolic profile with 20 distinct compounds identified. Among these, eucalyptol (1,8-cineole), a monoterpenoid ether, emerged as a prominent constituent. Singh et al. (2024) reported that 1,8-cineole has antifungal properties and is considered a potential alternative to synthetic fungicides against *Alternaria tenuissima.* At a minimum inhibitory concentration of 5 μL/mL, it was found to significantly reduce ergosterol synthesis, accompanied by impaired cell membrane function, sprouting of the germ tube, and reduced cell wall rigidity. The presence of phenylethyl alcohol, an aromatic alcohol with established antifungal activity, further supports the biocontrol potential (Etschmann et al., 2002). Zou et al. (2022) demonstrated that exposure to 2-Phenylethanol inhibits the growth of *Botrytis cinerea* through oxidative stress and cell membrane disruption. *T. harzianum* 404.1 also produced 1,2-Benzenedicarboxylic acid, butyl 2-ethylhexyl ester, a potent volatile organic compound that exhibits properties that are anti-inflammatory, cytotoxic, antifungal, and mycolytic in nature (Dey et al., 2025). Shabanamol et al. (2021) stated that the compound has strong fungal-degrading activity, as evidenced by the dispersal and shrinkage of mycelia of the pathogen *Rhizoctonia solani.* The compound hexanedioic acid, bis(2-ethylhexyl) ester, is a plasticizer derivative (Ghisari and Bonefeld-Jorgensen, 2009). Its effectiveness against several types of fungi, including *Fusarium* sp., has been reported (Ferdosi et al., 2021). A survey of the literature has revealed that the hexanedioic acid compounds exhibit a range of properties, including antimicrobial, anti-inflammatory, larvicidal, and anticancer effects (Rehman et al., 2019).

*T. longibrachiatum* (U-41) exhibited a smaller metabolic profile with 15 compounds, several of which overlap with those of other species. This isolate shared with *T. harzianum* 404.1 (U-53) phenylethyl alcohol and hexanedioic acid, bis(2-ethylhexyl) ester. Also, this species produced 9,10-anthracendione, 2,7-dimethyl- (2,7-dimethylanthraquinone), as anthraquinones, which are known chemical constituents in the *Trichoderma* (Siddiquee, 2014). Anthraquinones have several pharmacological activities and have been found to act as immunostimulants, antifungals, antivirals, and anticancer agents (Siddiquee, 2014; Zhao and Zheng, 2023).

*T. asperellum* (J-3) produced 18 compounds with a notable emphasis on oxygenated terpenoids and aromatic ketones. Bicyclo[3.1.0]hex-2-ene, 2-methyl-5-(1-methylethyl)- is an alpha-thujene compound (Ishak et al., 2024). Alpha-thujene compounds extracted from the essential oils of *Boswellia serrata* exhibit high activity against *Trichophyton* spp., a dermatological pathogen (Abd Rashed et al., 2021). Caryophyllene oxide, an oxygenated terpenoid, also stands out (Yang et al., 2000). Numerous studies have demonstrated its antifungal activity, specifically β-Caryophyllene oxide’s ability to inhibit the growth of *Fusarium* spp. This is achieved by suppressing ribosome synthesis and function, as well as inhibiting the overall growth of this pathogen (Liao et al., 2024). Jassal et al. (2021) found that caryophyllene and caryophyllene oxide have antifungal activity against several fungi, including *Fusarium moniliforme, Rhizoctonia solani,* and *Helminthosporium oryzae* (Jassal et al., 2021). Also, the biologically active compound 1,2-Benzenedicarboxylic acid, butyl 2-methylpropyl ester, was detected from *T. asperellum* (J-3). According to a study by Lu et al. (2018), the presence of 1,2-benzenedicarboxylic acid, butyl 2-methylpropyl ester, and (Z)- 9-octadecenamide from the liquid fertilizer extracted with ethyl acetate was detected, and this extract achieved up to 84% inhibition of the pathogen *F. oxysporum*.

*T. koningii* (U-27) produced 16 compounds with several unique metabolites not detected in other species. The highest peak in the GC–MS chromatogram analysis revealed the compound 2H-Pyran-2-one,6-pentyl- (6PP). 6-PP represents a significant volatile organic compound that is biosynthesized by various species of *Trichoderma (*Garnica-Vergara et al., 2015). Numerous studies have demonstrated that this compound has antifungal properties, particularly against plant pathogenic fungi (Wu et al., 2023). It can also stimulate the plant’s defenses against pathogenic fungi and promote growth (Xiao et al., 2023). In addition, this isolate shares with *T. harzianum* 404.1 (U-53) 1,2-Benzenedicarboxylic acid and butyl 2-ethylhexyl ester, whose properties were mentioned previously.

Despite the strong antifungal properties of the VOCs found in this experiment, the possible phytotoxic effect of some VOCs on *C. arabica* requires attention. For example, phthalate derivatives such as 1,2-benzenedicarboxylic acid butyl 2-ethylhexyl ester, which was detected in *T. harzianum* 404.1 and *T. koningii* U-27, show both cytotoxic and anti-inflammatory properties (Dey et al., 2025; Ghisari and Bonefeld-Jorgensen, 2009). In addition, anthraquinones like 2,7-dimethylanthraquinone, which is found in *T. longibrachiatum* U-41, can show pharmacological actions as well as phytotoxic effects at high concentrations (Zhao and Zheng, 2023). However, the concentration of these VOCs that *Trichoderma* will produce in the rhizosphere would be much lower compared to the concentration of pure compounds, and probably the soil will help to prevent any adverse effects. Anyway, in future research, it is necessary to measure the phytotoxic effect of these VOCs on coffee seedlings in greenhouse experiments and calculate such characteristics as root length, leaf area, and chlorophyll content at different levels of VOC concentration.

The GC–MS analysis carried out on four species of *Trichoderma* showed marked metabolic diversity, each species secreting a distinct set of volatile organic compounds. The detection of bioactive molecules such as terpenoids, aromatic alcohols, ketones, and polycyclic compounds has given molecular evidence on the role of these fungi as biocontrol agents. Thus, the evidence obtained supports the fact that *Trichoderma* strains have the potential to be used as biocontrol agents because they secrete diverse secondary metabolites. This mechanistic partitioning of plant pathogens by biocontrol fungi and plant roots has implications for strain selection and for the formulation of biocontrol fungi for practical use. First, since growth rate accounts for the majority of the variance in inhibition data, the simplest characteristic to measure in a screening program—growth rate—is also the most relevant for root colonization in the field (Cook, 1993). A simple dual culture assay may thus be used as a first screen to identify effective strains, which can then be further tested for biocontrol efficacy in more complex rhizosphere environments. Second, the potent marker caryophyllene oxide for *F. brachygibbosum* inhibition allows for the use of GC–MS for rapid screening of effective *Trichoderma* strains for biocontrol of this species. The differential levels of inhibition of the two Fusarium species by *T. asperellum* and *T. harzianum* suggest that a single biocontrol strain is unlikely to provide broad-spectrum control of root pathogens; a consortium of strains may be more effective. While the usage of consortia derived from Trichoderma species is beneficial in controlling plant diseases such as *Phytophthora ramorum* in forest nurseries (Xiao et al., 2023), the usefulness of these technologies in relation to the challenge at hand has yet to be proved. A consortium consisting of *T. asperellum* J-3 producing caryophyllene oxide, *T. koningii* U-27 producing 6-PP, and *T. harzianum* 404.1 would, in our judgment, have a synergistic impact. Such consortia of biocontrol fungi are consistent with the expected diversity of fungal species in the rhizosphere, where *Trichoderma* spp. are known to occur in complex communities (Guo et al., 2019). Finally, the volatile nature of the biocontrol compounds (1,8-cineole, boiling point 176 °C; 6-PP, boiling point 235 °C) identified here as having biocontrol activity against root pathogens, suggests that the most effective method for their delivery to the root zone would be via soil incorporation, since these compounds are highly volatile at temperatures near to the boiling point of water and thus would rapidly dissipate from the foliar surface of roots growing n soil (Yang et al., 2000).

In addition to being geographically innovative with respect to Saudi Arabia, the scientific novelty of this current research paper lies in three specific mechanistic contributions. First, quantifying the variance in inhibition of the *Fusarium* pathogens by each of the three mechanisms of action through hierarchical regression analysis, which has never been used in a study on *Trichoderma-Fusarium* interactions before. Second, the detection of the correlations specific to species for the individual volatiles with respect to pathogen-specific inhibition, where caryophyllene oxide proved to be highly correlated with *F. brachygibbosum* inhibition, and 6-pentyl-2H-pyran-2-one was correlated with *F. oxysporum* inhibition. Third, the finding that growth rate competition accounted for over 50% of the variance in inhibition thus allows one to base the selection of strains not only on antagonism. These mechanistic findings have immediate implications for the rational development of biocontrol formulations.

## 5 Conclusion

In conclusion, this study has three major messages for *Trichoderma* biocontrol of coffee *Fusarium* root disease. (1) Growth (competition) is the major mechanism of biocontrol by *Trichoderma* spp. and that of *Fusarium* spp. recorded in this study are 5.9–7.9 mm/day, while those of *Trichoderma* spp. are 15–19 mm/day (50% + of the variance in antagonism *in vitro*). (2) VOCs that *Trichoderma* spp. produce and affect molecular targets, including ergosterol synthesis (1,8-cineole), ribosome function, TOR signaling pathway (6-PP), and membrane integrity (2-PE), which affect an additional 22–25% of the variance in antagonism in vitro (pathogen-specific). (3) Mycoparasitism of *Trichoderma* spp. towards *Fusarium* spp. as observed by light and scanning electron microscopy is not predictive of inhibition in this study, implying that Trichoderma biocontrol of root infections of coffee caused by *Fusarium* spp. is primarily a matter of competition for space and/or influence of growth, followed by and/or affected by VOCs emitted by *Trichoderma* spp. before contact.

Although the study was optimized to allow maximum production of VOCs, in field conditions, VOCs will experience several limitations, including diffusion, adsorption onto soil particles, and degradation by other microorganisms in the soil. Therefore, future research should focus on determining the minimum inhibitory concentrations of individual VOCs against *F. oxysporum,* testing the predictions of the current model in a greenhouse study using natural soils, and determining the emission rates of VOCs from *Trichoderma-colonized* roots of coffee seedlings using solid-phase microextraction. In addition, testing the predicted benefits of using a consortium of *Trichoderma* strains in plot field trials conducted across Jazan, Al-Baha, and Asir provinces.

Relying on the aforementioned findings, we recommend using *T. harzianum* 404.1 and *T. aspeeellum* J-3 for the control of *F. brachygibbosum*-dominant root diseases of coffee. Both isolates exhibit superior inhibition by the VOCs of the two isolates and provided more than 77% inhibition in the in vitro biocontrol assays. For control of *F. oxysporum*-dominant root diseases, we recommend the use of *T. koningii* U-27, as it is the only isolate able to produce 6-PP in culture. For mixed infections, the use of a consortium of the three best isolates (*T. harzianum 404.1, T. asperellum J-3,* and *T. koningii U-27*) may offer complementary mechanisms in controlling root diseases of coffee. That hypothesis might require an experimental evaluation and field trials of the selected *Trichoderma* isolates. Future studies to evaluate the consortium behavior against mixed infection should be conducted and to assess potential synergistic or antagonistic interactions between the Trichoderma strains under optimal conditions.

## Data Availability

The datasets presented in this study can be found in online repositories. The names of the repository/repositories and accession number(s) can be found in the article/Supplementary material.

## Footnotes

1
https://dna.macrogen.com

2
https://blast.ncbi.nlm.nih.gov/Blast.cgi

3
https://tree.bio.ed.ac.uk/software/figtree/
